# The Loss of Myocardial Benefit following Ischemic Preconditioning Is Associated with Dysregulation of Iron Homeostasis in Diet-Induced Diabetes

**DOI:** 10.1371/journal.pone.0159908

**Published:** 2016-07-26

**Authors:** Vladimir Vinokur, Sarah Weksler-Zangen, Eduard Berenshtein, Ron Eliashar, Mordechai Chevion

**Affiliations:** 1 Department of Biochemistry and Molecular Biology, Institute of Medical Research Israel-Canada, The Hebrew University of Jerusalem, Jerusalem, Israel; 2 The Diabetes Unit, Department of Internal Medicine, Hadassah-Hebrew University Medical Centre, Jerusalem, Israel; 3 Department of Otolaryngology-Head and Neck Surgery, Hadassah-Hebrew University Hospital, Jerusalem, Israel; CINVESTAV-IPN, MEXICO

## Abstract

Whether the diabetic heart benefits from ischemic preconditioning (IPC), similar to the non-diabetic heart, is a subject of controversy. We recently proposed new roles for iron and ferritin in IPC-protection in Type 1-like streptozotocin-induced diabetic rat heart. Here, we investigated iron homeostasis in Cohen diabetic sensitive rat (CDs) that develop hyperglycemia when fed on a high-sucrose/low-copper diet (HSD), but maintain normoglycemia on regular-diet (RD). Control Cohen-resistant rats (CDr) maintain normoglycemia on either diet. The IPC procedure improved the post-ischemic recovery of normoglycemic hearts (CDr-RD, CDr-HSD and CDs-RD). CDs-HSD hearts failed to show IPC-associated protection. The recovery of these CDs-HSD hearts following I/R (without prior IPC) was better than their RD controls. During IPC ferritin levels increased in normoglycemic hearts, and its level was maintained nearly constant during the subsequent prolonged ischemia, but decayed to its baseline level during the reperfusion phase. In CDs-HSD hearts the baseline levels of ferritin and ferritin-saturation with iron were notably higher than in the controls, and remained unchanged during the entire experiment. This unique and abnormal pattern of post-ischemic recovery of CDs-HSD hearts is associated with marked changes in myocardial iron homeostasis, and suggests that iron and iron-proteins play a causative role/s in the etiology of diabetes-associated cardiovascular disorders.

## Introduction

Cardiac ailments constitute leading causes of morbidity and mortality in Western world [1,2]. Efforts are continuously being invested in trials to protect the ailing heart. Murry *et al*. and subsequent publications on heart patients and animal models of heart ischemia and reperfusion demonstrated that ischemic preconditioning (IPC)–subjecting the heart to a series of brief periods of ischemia separated by short periods of perfusion–markedly improved the post ischemic functional recovery and the heart capacity to withstand a subsequent, more intensive insult. Indeed, the IPC procedure was employed clinically during and following cardiac surgery, percutaneous transluminal coronary angioplasty (PTCA), and heart transplantation [3]. Angina pectoris (chest pain), a clinical analog of experimental IPC, if experienced immediately prior to acute myocardial infarction (AMI) has proven protective to the human heart [3,4]. Nevertheless, the beneficial effect of IPC on *diabetic hearts* is still controversial [5,6].

Iron is an essential element in all cells and tissues. Excess of labile iron can prove detrimental and cause tissue injury. Similar double-faceted behavior has already been identified for the effects of reactive oxygen-derived species (ROS). Low levels of ROS often serve as cellular messengers, indispensable for normal function, while high concentrations are deleterious. Labile and redox-active iron is a key participant in ROS-induced tissue-injury, including inflammation [7], through the catalysis of the production of highly reactive species, like the hydroxyl radical. We have recently demonstrated [8,9,10] that cardiac IPC initiates marked *de novo* synthesis of ferritin, which in turn, serves as a 'sink' for high levels of ischemia-induced released iron ions, thus protecting the heart against iron-mediated I/R damage. Also, we have shown, that ferritin level in naïve (non-manipulated) hearts from Type-1-like streptozotocin (STZ)-induced diabetic rats was 2-fold higher than in non-diabetic controls [8]. This was in accord with the enhanced resistance of the diabetic heart to I/R injury when compared to controls. During IPC ferritin level increased markedly. Unlike in the controls, the diabetic hearts failed to maintain the high ferritin levels during the subsequent ischemia phase, which explained the loss of the cardio-protective benefits of IPC, when reperfused.

In this study we focused on diet-induced Type 2-like diabetes. The Cohen-Diabetic rat model, employed in this study, comprises two genetically derived contrasting strains. The sensitive-CDs-strain that develops hyperglycemia when fed on a diabetogenic low-copper-high-sucrose-diet (HSD) but maintains normoglycemia on regular diet (RD). The resistant-CDr-strain does not develop hyperglycemia even when fed on HSD [11]. Hyperglycemic-CDs-rats show normal fasting glucose levels without insulin resistance but their pancreatic β-cells exhibit markedly reduced capacity to secrete insulin in response to glucose (GSIS) [11,12]. Un-like the STZ-induced Type 1 diabetic model, all the CDs-rats exhibit Type-2-like diabetes phenotype, without intermediate groups characterized by lower blood glucose levels.

A tight association between copper availability and iron homeostasis has been well established [13]. A number of proteins, like hephaestin and ceruloplasmin, are involved in cellular uptake and secretion of iron. These proteins contain essential copper ions in their active center or their prosthetic group. Thus, a copper-deficient diet may prove detrimental for the maintenance of adequate iron homeostasis, on the cellular and whole organism levels, leading to a variety of disorders [13].

## Materials and Methods

### Animals

Eight-week-old male CDs and CDr rats fed RD were switched to HSD for a period of 30 days. Controls were maintained on RD for the same time period. Rats were divided to 4 subgroups: CDs-RD, CDr-RD, CDs-HSD and CDr-HSD; only the CDs-HSD rats turned hyperglycemic [11]. The average body weight were as following: CDs-RD—263±18g; CDs-HSD—262±12g; CDr-RD—260±7g; CDr-HSD—259±12g. The values are shown as average ± standard error. Hyperglycemia was confirmed in CDs-rats after 30 days on the HSD by a 2 hr postprandial blood glucose test of >200 mg/dl [12,14]. Blood glucose values of the other groups were 112±4 mg/dl in CDr-HSD group, 94±6 in CDs-RD, and 105±4 in CDr-RD (The values are shown as average ± standard error). The rats were bred and maintained in the animal facility at the Hebrew University School of Medicine, Jerusalem, Israel, under standard conditions (12 h light/12 h dark) and fed an appropriate diet and distilled water *ad libitum*.

The experimental protocols had been approved by the Institutional Animal Care and Use Committee of the Hebrew University of Jerusalem (MD-13-13922-3).

### Diet

RD consisted of a mixture of ground whole wheat, ground alfalfa, bran, skimmed milk powder, and salts, resulting in 21% protein, 60% carbohydrates, 5% fat, and 0.45% NaCl content (Koffolk, Israel). Custom prepared HSD contained 72% sucrose; 18% vitamin-free casein; 5% salt mixture No 2I XIII (MP Biomedicals, USA); 4.5% butter; 0.5% corn oil, vitamins and low copper (0.9 ppm) [11,15].

### Blood glucose level

At 12 weeks of age blood glucose level (BG) was determined at 2h postprandial. CDs-RD, CDr-RD and CDr-HSD rats maintained BG within the normal range (<140 mg/dl), while CDs-HSD exhibited high BG (>200mg/dl).

#### Heart perfusion and hemodynamic parameters

Prior to terminal surgery of heart excision the rats were injected with sodium heparin (500 units/kg i.p.), followed after 30 min by a mixture of 80 mg/kg ketamine and 5 mg/kg xylazine 100 μl i.p. Upon bilateral thoracotomy, hearts with a segment of the ascending aorta were rapidly removed, and put for 5 min. in ice-cold heparinized saline. The average weight of the heart was 1.1±0.1 g. No differences between the groups were detected.

The perfusion of the isolated rat heart was performed on Langendorff apparatus as described previously [8,9], using a #3 balloon (Harvard Apparatus, MA, USA). The perfusion buffer was a "blood-free" solution of modified Krebs-Henseleit (KH) buffer. It contained 118 mM NaCl, 5.8 mM KCI, 2.5 mM CaCl_2_, 1.2 mM MgSO_4_, 25 mM NaHCO_3_, and 11.1 mM glucose. All perfusion buffers were prepared the day of the experiment in double distilled water and filtered prior to use. All vessels were prewashed with EDTA (0.1 M) before use. The temperature within the system had been constantly maintained at 37°C. Heart rate (HR), end diastolic pressure (EDP), developed pressure (DP), and its derivatives (+dp/dt and -dp/dt) were recorded and evaluated. At the beginning of the experiment the EDP value was set manually as 0 mm Hg. All primary data was processed using a customized version of LabView software. Work index (WI = Heart Rate x DP) was used as a measure of heart contractility. The degree of cardioprotection was expressed by the percent ratio of two values: WI at the 120^th^ minute (completion of the reperfusion phase) and WI at 10^th^ minute (the last minute of the stabilization phase). At the end of the experiment the heart was snap-frozen in liquid nitrogen and stored at -80°C for further analysis.

### Experimental protocols

**Fig 1:**
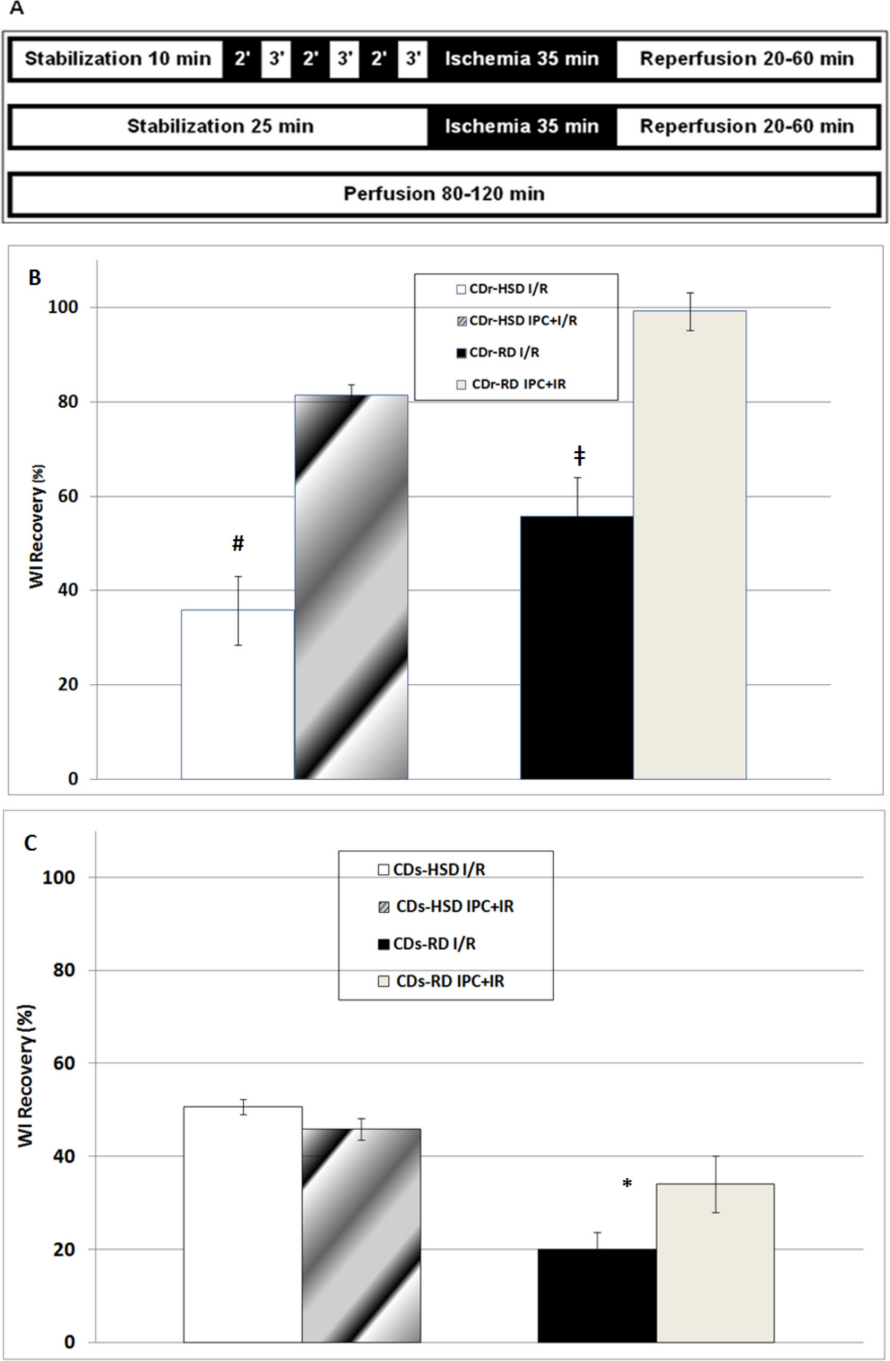
**Three basic experimental protocols (A) and post-ischemic recovery of the WI (B and C).** (A) (i) IPC followed by I/R (upper bar); ii) I/R (middle bar); and (iii) continuous perfusion (lower bar). (B and C) Post-ischemic recovery of WI for hearts from Cohen diabetes-resistant (CDr) rats (Panel 1B) and Cohen diabetes-sensitive rats (CDs) (Panel 1C), subjected to I/R without and with prior IPC. Animals were fed on either high sucrose/low copper diet (HSD) or regular (RD) diets. WI was calculated as the product of the Developed Pressure x Heart Rate (DP*HR). The degree of cardioprotection was expressed by as percent (%) ratio of two values: WI at the 120^th^ minute (completion of the reperfusion phase) and WI at 10^th^ minute (the last minute of the stabilization phase). Mean ± SEM values are shown; *—p<0.05 in IPC+I/R *versus* I/R in CDs-RD; ^**#**^—p<0.05 in IPC+I/R *versus* I/R in CDr-HSD; ^**‡**^—p<0.05 in IPC+I/R *versus* I/R in CDr-RD.

The three basic experimental protocols often used in our lab [8] were employed (Fig 1A): (i) IPC followed by I/R (upper bar); (ii) I/R (middle bar); and (iii) continuous perfusion (lower bar). For each time-point, 7–9 hearts were tested.

### Tissue homogenates

were prepared using a Teflon homogenizer (Cole Parmer, USA), homogenizing ~50 mg wet tissue, which had been excised from the right part of the apex of the freshly defrosted heart in 1 ml of the lysis buffer. After centrifugation at 10,000xg for 5 min the pellet was discarded, and protein concentrations in the supernatant were determined using BCA kit, according to the manufacturer's instruction (Pierce, USA).

### Ferritin (Ft) levels

in tissue homogenates were determined using an ELISA-based "sandwich" assay [9,16]. The antibodies against L or H rat Ft were prepared developed as described earlier [9]. Briefly, the goat anti-rat liver-Ft was diluted in coating buffer and 200μl /well were used to coat 96-well micro-ELISA plates (Nunc, Roskilde, Denmark). After washing of the blocked plates with washing buffer, 200 μl of samples or standards diluted in dilution buffer were applied to the wells, followed by 1-h incubation at 37°C. After further washings as above, 200 μl /well of Rabbit anti-rat heart ferritin in same dilution buffer, was added and incubated for 1 h at 37°C. Next, after further washings, 200 μl of goat anti-rabbit γ-globulin-antibodies, conjugated to β-galactosidase in conjugate buffer was added and incubated for 1 h at 37°C. Then, following washings as above, 200μl /well of chlorophenol-red β-*d*-galactopyranoside (Roche diagnostic GmbH, Manhiem, Germany) diluted in substrate buffer was added and incubated until color was obtained. The developed color was read in a microplate reader (MR 5000 Dynatech Laboratories, Chantilly, VA, USA). A primary filter with a peak transmission at 570 nm and a secondary filter with a transmission at 620 nm were used. [8,16,17,18].

### Ferritin-bound iron

**Fig 2:**
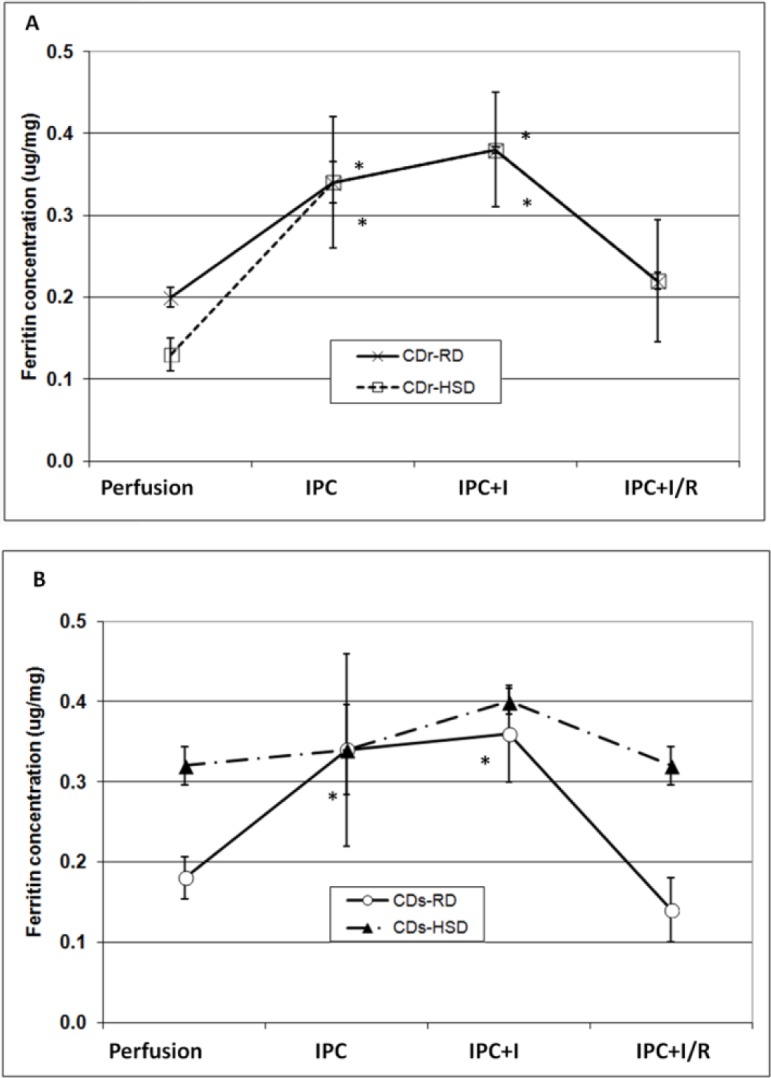
Ferritin levels in hearts of Cohen rats. Ferritin levels in hearts from Cohen diabetes-resistant rats (CDr) (Panel A) and Cohen diabetes-sensitive (CDs) (Panel B), fed on either high sucrose diet (HSD) or regular diet (RD) and subjected to IPC+I/R protocol. In these experiments hearts were stabilized (10 min), followed by the IPC procedure (15 min), ischemia (35 min) and reperfusion (60 min). Ferritin concentration was measured using ELISA. Means ± SEM are shown. *—denotes p < 0.05 *versus* Perfusion for the same subgroup.

Ferritin was immuno-precipitated from hearts homogenate, using a mixture of anti-H and anti-L ferritin antibodies, according to previously described procedure [9,16,17,18] as following. Aliquots of the tissue homogenate containing at least 1 mg of ferritin (calculated according to the results of ELISA assay—see Fig 2) were incubated at 70°C for 10 min., chilled on ice, centrifuged at 14000 g for 30 min and the pellet was discarded. The supernatant was mixed with anti-rat heart ferritin antibodies and incubated for 72 h at 4°C. Then the samples were centrifuged at 20,000 g for 20 min, the supernatant discarded and the pellet dissolved in 100 μl HNO_3_ at 37°C by overnight incubation [9,16,17,18]. Iron content was determined by Zeeman atomic absorption spectrometry [8].

### qRT-PCR measurements

were performed using previously published protocols [19,20]. Primers for the target genes and the housekeeping gene (β-Actin), used for normalization, were designed using Primer3 software (from: http://frodo.wi.mit.edu/cgi-bin/primer3/primer3_www.cgi) (S1 Table).

### Statistical analysis

The data was analyzed using repeated one-way ANOVA followed by the Scheffé post-hoc test for multiple comparisons (α = 0.05). Statistically significant differences were considered when p ≤ 0.05.

## Results

### Hemodynamic data

The hemodynamic parameters of rats hearts, which had been subjected to I/R with or without prior IPC, are shown in Table 1. When hearts of CDr rats, fed on either RD or HSD diet, were subjected to IPC+I/R, the recovery of the work index (WI), calculated as DP x HR at the end of the reperfusion phase, was better than for hearts subjected to I/R alone (Table 1; Fig 1B). CDs hearts showed an IPC-associated benefit only when fed on RD. The CDs-HSD hearts were not protected by the IPC procedure. Interestingly, WI recovery of CDs-HSD hearts following I/R (without prior IPC) was better (p<0.05) than of CDs-RD hearts (Fig 1C). This finding is in accord with the notion that these hearts are under continuous chronic partial preconditioning [8,21]. In summary, the functional protection, afforded to the hearts by IPC, was attained in the three normoglycemic groups, but not in the diabetic group.

**Table 1:** Hemodynamic parameters of the rats' hearts before and after I/R with and without prior IPC.

Group	Protocol	DP_0_ (mmHg)	HR_0_ (min^-1^)	DP_120_ (mmHg)	HR_120_ (min^-1^)	EDP _120_ (mmHg)
CDR-RD	I/R	94±5	231±12	61±12	199±8	33±17
	IPC+I/R	91±2	254±26	90±12*	259±23*	15±10
CDR-HSD	I/R	137±12	268±7	55±12	243±9	33±4
	IPC+I/R	125±19	238±13	99±11*	243±5	10±4*
CDS-RD	I/R	147±30	228±10	26±2	247±55	82±8
	IPC+I/R	156±9	217±15	51±13*	214±6	54±11*
CDS-HSD	I/R	145±12	236±7	77±9	228±13	52±2
	IPC+I/R	166±18	235±5	80±12	225±12	39±10

The basal and the final hemodynamic parameters (DP, HR and EDP) of the hearts subjected to I/R with or without prior IPC are shown. The following abbreviation are used: DP_120_, HR_120_ and EDP_120_– developed pressure, heart rate and end diastolic pressure, respectively, at the completion of the experiment—120 min

DP_0_ and HR_0_– developed pressure and heart rate at the stabilization phase

Mean±SEM are shown *p≤0.05 vs. I/R data in the matching group

### Ferritin concentrations

Hearts from either of the three normoglycemic groups (Fig 2), when subjected to IPC+I/R, showed analogous trends: a marked rise in ferritin concentration during IPC, further milder increase during the subsequent prolonged ischemia phase, and rapid degradation of ferritin and return to its basal level during the following reperfusion. In the hyperglycemic hearts the basal ferritin level was significantly higher (0.32±0.02 μg/mg) than the baseline level of the other three heart groups (0.17±0.02 μg/mg; p<0.05). Hearts subjected to I/R without prior IPC or to continuous perfusion maintained constant ferritin levels during the entire experimental protocols (data not shown).

### Ferritin saturation with iron

**Fig 3:**
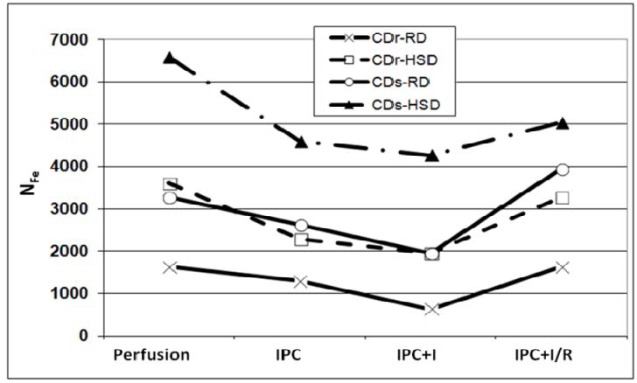
Ferritin saturation with Fe. Ferritin saturation with Fe (N_Fe_) in hearts of Cohen diabetes-sensitive and diabetes-sensitive rats fed on high-sucrose or regular diets, subjected to IPC and followed by I/R.

The average number of iron atoms contained within a single ferritin molecule (N_Fe_), which also indicates the degree of saturation of ferritin with iron, was monitored along the experimental protocols (Fig 3). For normoglycemic CDr-RD hearts N_Fe_ was between 1700 and 600 Fe atoms/molecule. Hearts of the other normoglycemic groups—CDs-RD and CDr-HSD showed higher N_Fe_ values, similar to each other (2900–4000 iron atoms/molecule), than those of CDr-RD group. These N_Fe_ values remain within the normal range reported in tissues (up to 4500 atoms/molecule) [22].

The experiments were conducted using the isolated rat heart (Langendorff) model. Along the protocols no import and/nor a significant loss of iron from the heart occurred [9]. It is expected that the changes in N_Fe_, along the protocol, should be inversely related to the changes in ferritin levels, as was demonstrated for non-diabetic hearts [8].

In CDs-HSD hearts N_Fe_ values were markedly beyond the normal range (reaching ~6600 atoms/molecule at baseline), which is in accord with the often observed diabetes-associated iron overload [23].

### mRNA levels of ferritin subunits

**Fig 4:**
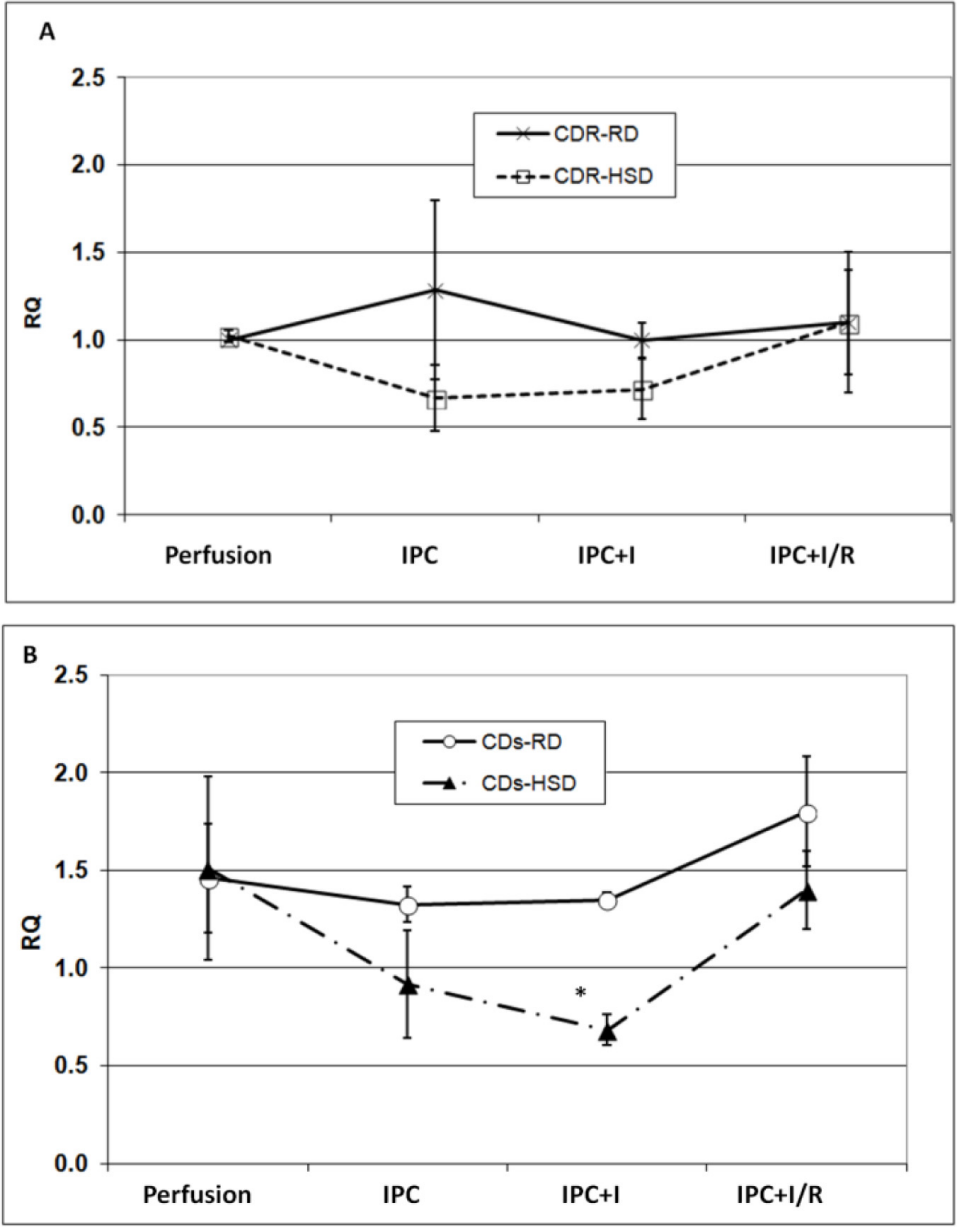
The levels of H-ferritin mRNA in Cohen rats subjected to IPC+I/R protocol. The levels of ferritin H-subunit mRNA (RQ) in hearts of Cohen diabetes-resistant and Cohen diabetes-sensitive rats' (A and B, respectively) when fed on HSD or RD, and subjected to IPC followed by I/R. Actin was used as a house-keeping gene. In the current experiments the measurements were performed at the end of the stabilization phase (10 min), followed by the end of IPC procedure (+15 min), completion of ischemia (+35 min) and end of reperfusion (+60 min). mRNA levels of H-subunit of ferritin were quantified by qRT-PCR. Means ± SEM are shown. *—denotes p < 0.05 *vs*. Perfusion (baseline) value of the same group.

The levels of mRNA of ferritin L-subunit, at baseline, were similar to each other in all four groups. Also, these mRNA values did not change throughout the entire IPC+I/R protocols (data not shown). mRNA levels of ferritin H-subunits was 1.5-fold higher in CDs hearts, when compared to CDr hearts (Fig 4). For CDr-RD, CDr-HSD and CDs-RD hearts mRNA levels of ferritin H-subunit remained constant along the entire IPC+I/R protocol. In contrast, in the diabetic CDs-HSD hearts, H-subunit mRNA levels decreased by 1.5-fold, during the IPC phase, and decreased further during the subsequent ischemia; the levels were restored to the baseline values during reperfusion.

## Discussion

Our previous study on iron homeostasis and Type-1-like STZ-induced diabetes in rat hearts [8] suggested a novel explanation for the diabetes-associated loss of the IPC response and the improved tolerance of the heart toward I/R injury. The basal level of ferritin—the protein crucial for sequestration and detoxification of labile and redox-active iron—in STZ-diabetic hearts was markedly higher than in non-diabetic hearts. Ferritin levels increased further to higher values during the IPC procedure, but failed to maintain these high levels during the subsequent prolonged ischemia phase.

In this study we investigated the physiology and iron homeostasis in hearts of genetically modified and diabetic-prone Cohen rats. Cohen rats develop diet-induced Type 2-like diabetes when fed on high-sucrose and low-copper diet (CDs-HSD). In previous studies by Weksler-Zangen S. et al. it was shown that hyperglycemia in the CDs-rat resulted from the combined effects of a genetic predisposition and the low-copper-level in the HSD diet. The genetic profile of the CDr rats protected them and enabled the maintenance of adequate glucose-stimulated insulin secretion (GSIS), despite the low-level of copper in the HSD diet, while the genetic diabetic sensitive rat—CDs—were susceptible to the HSD diet that initiated Type-2-like diabetes [11,12,14]. Efficient utilization of dietary iron in mammalian systems is supported by ceruloplasmin and hephaestin—two copper-containing proteins that function as a ferroxidases, oxidizing ferrous iron in a compartment for subsequent uptake [12]. Thus, copper deficiency could impairs normal iron metabolism. Signs of iron deficiency anemia appear in rats fed on a copper-deficient diet, and include low hemoglobin, low hematocrit and low red blood cells count [12,24,25]. Moreover, copper is required for the function of over 30 proteins, including Cu/Zn-SOD, which demonstrated reduced activity in red blood cells of copper-deficient pigs, rats and humans [26]. Systemic copper deficiency leads to cellular iron deficiency, which in humans results in impaired heart function and myocardial hypertrophy. Nevertheless, these phenomena were not seen in rats fed on a diet containing marginal dietary copper levels for a short period of time [27]. It should be noted that Fig 2A demonstrates the basal level of ferritin in CDr-HSD rats' hearts initially lower than in CDr-RD group. This could be due to compromised ferroxidase activity, stemming from copper-deficient diet.

In the current study, the focus is on hyperglycemic CDs rats fed on a copper poor diet for 30 days, so no cellular iron deficiency was expected. Indeed, we have previously demonstrated that HSD diet, alone, did not cause either anemia [11], or hypertrophy of the heart [14], and the activity of Cu/Zn-SOD remained unchanged (unpublished data). Diabetes is well known for causing proteins glycation. Among these proteins, copper-containing glycated proteins were shown to lose their copper atoms and to readily undergo degradation, promoting intracellular iron accumulation [24,25]. Thus, in CDs-HSD hearts the hyperglycemia-induced degradation of copper proteins did not lead not to anemia, but rather to diabetes-associated iron overload [28].

In this study we showed that hearts from Cohen sensitive diabetic rats contained (at baseline) nearly double the amount of ferritin, when compared to any of the other three non-diabetic groups, at baseline (Fig 2A and 2B), and to non diabetic controls [8]. This high ferritin level in CDs-HSD hearts, remained almost unchanged during the entire course of the IPC+I/R experiment. In contrast, heart ferritin of each of the other rat groups, responded to IPC by doubling its ferritin content, presumably, in order to scavenge the just released redox-active iron (released during the ischemia phase). Also, the N_Fe_ values in CDs-HSD hearts were abnormally high (Fig 3), and persisted after the IPC procedure and the subsequent prolonged ischemia, indicating that these hearts are "iron-overloaded".

It has been reported that ferritin stability was impaired under copper deficiency, turning ferritin into water-insoluble iron-saturated hemosiderin, which in turn, can be recognized by anti-ferritin antibodies [29]. This may explain the lack of changes in the amounts of ferritin, after IPC and ischemia, and the high level of ferritin-saturation with iron, in the CDs-HSD hearts.

Excessive levels of tissue-iron were shown to play central roles in diabetes-associated complications, in several organs [23]. which suggest a causative relationship between iron-overload in the pancreas and the onset of diabetes. Indeed, there is an increased risk of developing diabetes in patients suffering from hemochromatosis, or other forms of pathological body-iron overload.

Considering diabetic patients and diabetic animals, a direct link has been indicated, among hyperglycemia-induced atherosclerotic processes, iron overload and reduced cardiac muscle blood flow [21,30]. Hence, it can be suggested that the diabetic heart is continuously under partial ischemia [31], and consequently under partial ‘chronic preconditioning’ [8,32]. This explains the observed enhanced tolerance of the myocardium to injury that is induced by prolonged ischemia (without prior IPC). It also explains the loss of the IPC-induced functional benefit, in diabetic hearts, in animal models and human subjects [5,32,33]. Nevertheless, the IPC response in the diabetic heart *and* the detailed mechanism/s underlying this response are still not fully understood. Our recent observations using the STZ-induced diabetic rat hearts [8] propose that iron homeostasis, as expressed by high ferritin levels, at the onset of the reperfusion phase played a crucial role in IPC-protection.

On the transcriptional level, a noticeable decrease in H-ferritin mRNA level was observed in CDs-HSD hearts after IPC+ischemia. No significant alterations in the levels of mRNA of ferritin were detected in any of the other groups (for H-ferritin—Fig 4; for L-ferritin—data not shown). The following explanation can be suggested: under normal conditions the ratio H/L between the amounts of cardiac ferritin subunits was 3:2, where H-subunit is the dominant [34]. During post-IPC-ischemia this ratio was altered in favor of L-subunit dominance, indicating production of L-rich ferritin, a protein that is extrinsic to the heart. L-subunit-rich ferritin has a higher affinity for iron than the H-subunit-rich protein [35]. Thus, the newly synthesized L-rich ferritin could sequester excessive amounts of iron more efficiently than the normal cardiac H-dominant ferritin, therefore preventing ROS formation and inhibiting oxidative stress [36,37].

In summary, the observations in this study demonstrate the generality of the roles played by iron and its proteins in IPC under diabetes. The previous study was centered on STZ-induced Type 1 diabetic model (8), while the current investigation focuses on the physiology and biochemistry of the nutrition-induced diabetes in susceptible Cohen rats. The current study further allowed the discrimination between two opposing powers acting in these hearts: (i) the diabetogenic effect of high sucrose diet and the diabetes-associated iron overload, and (ii) the copper induced processes that lead to iron deficiency. The data clearly showed that the diabetogenic process of the diet dominated the overall effect of HSD on CDs rat hearts.

## Supporting Information

S1 Table The primers' sequences and relevant exons numbers used in the PCR assay. The nucleotide sequences used for primer design were obtained from the public database GenBank. Primers for the indicated genes were constructed using the ‘Primer3’ software, and designed so that one of the primers in each pair was complimentary to the exon-exon boundary (e.g., 3–4) in order to avoid genomic DNA amplification [20].
(DOCX)
