# Supplementary material for: The Loss of Myocardial Benefit following Ischemic Preconditioning Is Associated with Dysregulation of Iron Homeostasis in Diet-Induced Diabetes
Source: PLoS One. 2016 Jul 26;11(7):e0159908. doi: 10.1371/journal.pone.0159908 (PMC4961428; doi:10.1371/journal.pone.0159908)

Supplemental Table 1.

**S1 Table. The primers' sequences and relevant exons numbers used in the PCR assay.**


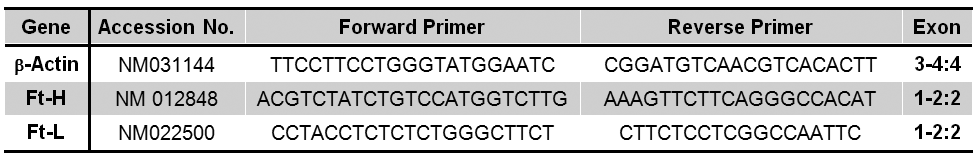

Supplement: S1 Table — The nucleotide sequences used for primer design were obtained from the public database GenBank. Primers for the indicated genes were constructed using the ‘Primer3’ software, and designed so that one of the primers in each pair was complimentary to the exon-exon boundary (e.g., 3–4) in order to avoid genomic DNA amplification [20]. (DOCX) [file pone.0159908.s001.docx]
